# Secure Base Leadership in military training: enhancing organizational identification and resilience through work engagement

**DOI:** 10.3389/fpsyg.2024.1401574

**Published:** 2024-12-24

**Authors:** María C. Navas-Jiménez, Ana Laguía, Patricia Recio, Carlos García-Guiu, Alberto Pastor, Sergio Edú-Valsania, Fernando Molero, Mario Mikulincer, Juan A. Moriano

**Affiliations:** ^1^Department of Social and Organizational Psychology, Faculty of Psychology, Universidad Nacional de Educación a Distancia (UNED), Madrid, Spain; ^2^Department of Methodology of Behavioral Sciences, Faculty of Psychology, Universidad Nacional de Educación a Distancia (UNED), Madrid, Spain; ^3^Joint Research Institute National University for Distance Education and Health Institute Carlos III (IMIENS), Madrid, Spain; ^4^Multidisciplinary Area, Centro Universitario de la Defensa, Zaragoza, Spain; ^5^Army Health Direction, Ministry of Defence, Madrid, Spain; ^6^Department of Social Sciences, Universidad Europea Miguel de Cervantes, Valladolid, Spain; ^7^Baruch Ivcher School of Psychology, Reichman University, Herzliya, Israel

**Keywords:** JD-R, Secure Base Leadership, work engagement, resilience, organizational identification, military leadership, military school, cadets

## Abstract

**Introduction:**

This study examines the relationships between secure base leadership, organizational identification, and resilience among military cadets, utilizing the Job Demands-Resources (JD-R) model as a theoretical framework. Specifically, it explores the mediating role of work engagement in these associations within the context of military training.

**Methods:**

A cross-sectional study was conducted with 363 cadets from the General Military Academy of the Army in Zaragoza, Spain. The sample comprised second-year cadets (*n* = 170; 46.8%) and third-year cadets (*n* = 193; 53.2%), with a gender distribution of 84% male and 16% female. Participants evaluated their section chief captains using the Leader as Security Provider Scale and completed validated questionnaires measuring work engagement, organizational identification, and resilience. Data were analyzed using partial least squares structural equation modeling (PLS-SEM) to test the hypothesized relationships and mediation effects.

**Results:**

The findings revealed that secure base leadership is positively associated with work engagement among cadets. Work engagement significantly mediated the relationships between secure base leadership and both organizational identification and resilience. The structural model explained a substantial proportion of variance in the outcome variables, supporting the applicability of the JD-R model in this context.

**Discussion:**

These results underscore the importance of secure base leadership in promoting work engagement, which in turn enhances organizational identification and resilience among military cadets. The study highlights the role of leaders as secure bases in fostering personal and organizational well-being. Implications suggest that incorporating secure base leadership principles in military training programs could contribute to the professional development and overall well-being of military personnel.

## 1 Introduction

Military academies are exceptionally challenging environments, requiring cadets to demonstrate extraordinary levels of discipline and engagement. Military training is known to be stressful, pushing cadets' physical and mental capacities to the limit and testing their endurance (Chen et al., 2022; Gibson and Myers, 2006; Myers and Bechtel, 2004). Within these institutions, cadets are also expected to strictly adhere to established codes of conduct and assimilate into a well-defined hierarchical structure. This integration often leads to a blurring of personal and professional life, making the clear delineation of boundaries a challenge (Soeters, 2018). It is within this context that cadets' work engagement becomes critical, especially as they navigate job demands where personal and professional realms are intertwined (Hall, 2011). The formal learning in the Military academies provide important role models and references where the character gets forged (Díez et al., 2023).

The Job Demands-Resources (JD-R) model (Bakker and Demerouti, 2007) offers a comprehensive framework for understanding individual variations in work engagement and its role in invigorating organizational identification and performance. This model suggests that the interplay between job demands and resources is key to driving employee motivation. Whereas, job demands may pose challenges for employees' motivation, the presence of adequate resources, such as social support and professional development opportunities, plays a significant role in enhancing work engagement. According to the JD-R model, work engagement thrives when employees are equipped with resources that effectively counterbalance their job demands, and heightened engagement fosters dedication, vigor, and absorption in their work. Within this framework, leadership is identified as a key resource (Mazzetti et al., 2023), particularly in military settings (Bates et al., 2013). Leadership not only influences the challenges faced by service members but also impacts their autonomy, available support, and commitment to their duties (Alarcon et al., 2010). Furthermore, research has shown that positive leadership styles, such as authentic leadership, are crucial in fostering work engagement within military environments (Moreno et al., 2021; Pastor et al., 2019).

Secure Base Leadership (SBL), drawing from attachment theory, represents a paradigm shift in positive leadership styles (Molero et al., 2019). This approach parallels the functions of attachment figures and emphasizes leader's provision of felt security (i.e., confidence that support will be available when needed) to subordinates. Effective leaders under this model are characterized by their keen responsiveness to and alignment with their members' needs, offering targeted guidance, emotional support, and encouragement. Secure Base Leadership plays a critical role in cultivating self-esteem, competence, autonomy, and resilience among organizational members. This fosters an environment where individuals are encouraged to embrace challenges and develop new competencies, catalyzing personal growth (Haslam et al., 2015). In the context of military academies, SBL is particularly effective in empowering cadets by providing a stable foundation of support. This support is essential for their full engagement in rigorous training and encourages risk-taking as an integral part of their development.

Hence, to support the implementation of SBL, our study aims to investigate the personal and organizational benefits of SBL within a military setting while examining the role that military cadets' work engagement play in mediating the contribution of SBL to two pivotal outcomes—organizational identification and resilience. Our primary objectives are to examine (a) how SBL, as a distinct job resource informed by attachment theory, enhances cadets' engagement in their roles and responsibilities within the military academy setting, and (b) whether this engagement contributes to cadets' organizational identification and resilience. We hypothesize that increased work engagement, nurtured by the supportive and empowering environment characteristic of SBL, will lead to a more profound organizational identification. Furthermore, we aim to explore the impact of this enhanced engagement, facilitated by SBL, on the development of resilience in cadets, enabling them to manage the inherent challenges and stressors of their rigorous training more effectively. By delving into these dynamics, our study seeks to contribute valuable insights into the transformative effects of SBL on cadets' professional growth and wellbeing, all within the JD-R model's conceptual boundaries.

## 2 Literature review

### 2.1 Work engagement

Work engagement (WE) is conceptualized as a positive, fulfilling, work-related state of mind that is characterized by vigor, dedication, and absorption (Schaufeli et al., 2002). Vigor reflects persistent energy and mental resilience while working, the willingness to invest effort in job tasks, and persistence even in the face of difficulties. Dedication is characterized by a sense of significance, enthusiasm, inspiration, pride, and challenge. Absorption is described as being fully concentrated and happily engrossed in one's work, whereby time passes quickly, and one has difficulties detaching oneself from work (Bakker, 2011). Work engagement is a persistent affective-cognitive state rather than a momentary state. It is not focused on a particular object, event, individual, or behavior (Salanova et al., 2005). Work engagement differs from job satisfaction in that it involves a dynamic state of work-related wellbeing that features not only a positive affective-motivational state of fulfillment (enthusiasm, pride, and inspiration) but also a high level of activation and energy (vigor, absorption; Bakker, 2011). This makes it a clearly motivational construct due to its elements of activation, energy, effort, persistence, and focused aim at achieving work-related goals.

Work engagement is fundamental for military personnel as it instills a deep-rooted commitment to superior performance. This psychological investment acts as a protective factor, enabling soldiers to withstand the challenges of military life, such as severe environmental conditions, separation from family, and restricted rest. Past findings indicated that soldiers with high levels of work engagement exhibit greater psychological robustness and show lower increases in stress under demanding situations, thereby highlighting the critical role of fostering work engagement for soldiers' welfare and proficiency in military operations (Britt and Bliese, 2003; Britt et al., 2004).

### 2.2 Secure Base Leadership

Secure Base Leadership (SBL) is a positive leadership style rooted in Bowlby's Attachment Theory (Bowlby, 1969) emphasizing the promotion of autonomy in subordinates, provision of supportive guidance during challenges, and nurturing of close, responsive relationships with followers. Hazan and Shaver (1990) initially noted that functions typical of attachment figures could be reflected in the workplace, with leaders serving as attachment figures for their employees. Expanding upon this foundation, Popper and Mayseless (2003) identified significant parallels between the characteristics of leaders and traditional attachment figures. They proposed that effective leaders, like caregivers that enhance their offspring's felt security, play an essential role in guiding, directing, and nurturing those who are less powerful or dependent on them. This includes being sensitive and responsive to the needs of organization members; providing advice, guidance, and emotional reassurance; assisting in the development of self-worth, competence, and autonomy; supporting the undertaking of new challenges; and encouraging personal growth (Haslam et al., 2015).

Building upon this groundwork, Molero et al. (2019) developed the Leader as a Security Provider Scale (LSPS) to evaluate subordinates' perceptions of their leaders as security-enhancing attachment figures within an organizational setting. The LSPS is based on three definitional criteria of a security-enhancing attachment figure: First, as a “secure base,” a leader supports and encourages followers to pursue goals within a safe environment while fostering their independence. Second, in the role of a “safe haven,” a leader provides calm, comfort, protection, and reassurance to followers during times of need. Third, “proximity maintenance” refers to a leader's ability to maintain a close relationship with followers, ensuring accessibility and minimizing the impact of separations. As a result, followers are more inclined to seek guidance, remain close to, and seek support from, their leader in challenging times, developing positive feelings toward them, and feeling protected in difficult situations.

Empirical evidence from studies employing the LSPS consistently shows a positive relationship between SBL and essential organizational and personal outcomes. These include enhanced work engagement, organizational citizenship behaviors, a positive psychological safety climate, and improved team performance (Lisá et al., 2021; Lisá and Greškovičová, 2023; Molero et al., 2019; Moriano et al., 2021). Additionally, SBL has been identified as a vital factor in reducing burnout and job stress (Lobato et al., 2023; Moriano et al., 2021), contributing to the maintenance of high levels of work engagement. The foundation of this relationship lies in the sense of safety and support SBL provides to employees, enabling them to perform optimally. In the absence of such support, employees may become consumed with concerns about threats to their wellbeing, which negatively impact their work output. In military contexts, leaders who enable environments that are psychologically safe (Lobato et al., 2023), in which subordinates can take reasonable risks without fear of retribution or negative consequences to self-image, status, or career, can increase engagement (Bates et al., 2013). Based on this evidence, we present the following hypothesis:

Hypothesis 1 (H1). Secure Base Leadership will be positively related to work engagement.

### 2.3 Organizational identification

Social Identity Theory examines how individuals perceive themselves as part of social groups (Tajfel, 2010; Turner, 1981). Central to this theory is the notion of organizational identification, a form of social identity that leads organization members to integrate their organizational membership and ties within their self-concept (Haslam, 2004). This integration is manifested both cognitively (e.g., internalization of organizational values) and emotionally (e.g., feelings of pride in being a member of the organization). In the military context, it is evident in the efforts of military institutions to foster a deep sense of unity among their members. These efforts are based on the anticipation that a strong identification with the organization will drive professional conduct, successful mission completion, and a higher likelihood of service members committing to long-term careers in uniform (Squires and Peach, 2020). This approach underscores the importance of a solid sense of belonging and adherence to military values in influencing personnel's attitudes and behaviors, thereby enhancing the effectiveness and unity of the military as an institution.

The transformation of cadets into officers within military academies is significantly shaped by their level of identification with the organization. Such identification cultivates a strong sense of belonging and commitment, essential in military environments that prioritize teamwork, discipline, and adherence to institutional values (Griffith, 2009; Topa et al., 2009). As this connection with the military deepens, cadets internalize its values, ethos, and standards of conduct, which are vital for effective leadership in challenging situations (Jennings and Hannah, 2011). This alignment not only fosters their organizational identity but also bolsters their capacity to lead with confidence and integrity, attributes that are indispensable for military officers. Therefore, the promotion of organizational identification during training is not merely advantageous but fundamental to nurturing competent and committed military leaders.

Secure Base Leadership (SBL) has been found to be positively associated with organizational identification, a key factor in the intention of military personnel to remain with the service (Squires and Peach, 2020). This leadership style, underpinned by its nurturing and supportive approach, seems to enhance the alignment of individual and organizational values. Molero et al. (2019) further substantiate this association by demonstrating that SBL not only strengthens organizational identification but also surpasses other positive leadership styles, such as authentic leadership, in achieving this aim. The effectiveness of SBL in fostering organizational identification is linked to its role in enhancing meaningful engagement, commitment, and the sense of challenge faced by members, thereby potentially improving their affiliation with the organization's goals. Based on this evidence, we formulate the following hypothesis:

Hypothesis 2 (H2). Secure Base Leadership will be positively related to organizational identification.

### 2.4 Resilience

Resilience research, whose roots can be found in the aftermath of the Second World War, was initially centered on the profound traumas experienced during that tumultuous period. In those times, resilience was understood as the ability to endure and thrive amidst a spectrum of adversities over a prolonged duration (Masten and Barnes, 2018). In the contemporary context, the prevalence of adverse and stressful situations in critical occupations such as the military (but also in emergency services and public safety professions) cannot be overemphasized (Bartone et al., 2007; Chérif et al., 2021). The nature of these workplaces exposes personnel to events and conditions that critically affect their wellbeing (e.g., extensive working hours, unusual schedules, dangerous tasks, and demanding environments).

Over time, resilience has been defined and conceptualized in multiple ways, especially within the behavioral sciences. In the context of this study, resilience is defined as the capacity to adapt to adversity or to recover from challenging circumstances (Bonanno et al., 2006). This definition is especially relevant to the military domain, where soldiers and officers are required to navigate not only the intrinsic challenges of military operations but also to endure and prevail in demanding and often austere deployment settings (Simmons and Yoder, 2013).

In military training, the emphasis on developing resilience in cadets transitioning to officers is vital. This stage of military education goes beyond mastering tactical skills and encompasses the cultivation of psychological resilience. Skills such as cognitive reframing, emotion regulation, and energy management are integral to this process. These skills, trainable and significantly influential, facilitate calm, solution-focused responses under stress (Zueger et al., 2023). By fostering resilience, cadets may be better equipped to manage the uncertainties and pressures of military life, rendering it a critical component not only for their initial training but also for their enduring effectiveness as military leaders who can adeptly navigate and excel in complex, high-stress environments (Chérif et al., 2021; Valor-Segura et al., 2020).

Studies involving military cadets suggest that leadership may influence subordinates' resilience, potentially guiding them to perceive and approach challenges with greater hardiness (Bartone et al., 2002, 2007). It is conceivable that SBL could play an important role in this aspect. Secure Base Leadership may facilitate the cognitive reframing of stressful experiences by empowering subordinates to perceive challenges as opportunities for growth and learning, with the assurance that support will be available when needed. In the context of military units, the ability of leaders to model and communicate positive reinterpretations of shared challenges could be crucial. Leaders who provide a secure base might be particularly influential under stressful conditions, possibly inspiring soldiers to see stressful events as manageable challenges that offer valuable learning opportunities. Given this potential, we propose the following hypothesis:

Hypothesis 3 (H3). Secure Base Leadership will be positively related to resilience.

### 2.5 Work engagement as a mediator

In the JD-R model, work engagement is a crucial mediating variable, linking job resources to positive outcomes both organizationally and personally. Job resources are believed to boost work engagement, which then enhances performance, job satisfaction, and wellbeing (Mazzetti et al., 2023). Leadership, including SBL, is typically viewed as a job resource in this model (Moriano et al., 2021). However, leadership might also be seen as an independent element within the JD-R model, as leaders can both reduce job demands and increase job resources. Thus, leadership might optimize working conditions for engagement by enhancing the positive effects of a work environment where cognitive demands and resources are both substantial (Decuypere and Schaufeli, 2020).

Applying this perspective to military academies, we argue that the supportive and empowering environment fostered by SBL significantly increases cadets' engagement with their roles. This heightened state of engagement may not only align their personal values and objectives more closely with those of the military organization but also cultivates a deep sense of belonging. This heightened sense of connection and identification with the organization is fostered by the positive experiences and satisfaction derived from being engaged in meaningful and fulfilling work supervised by a security-enhancing leader. Furthermore, meta-analytic studies have shown a positive relationship between work engagement and organizational commitment, a construct closely related to organizational identification (Mazzetti et al., 2023). Therefore, we propose the following hypothesis:

Hypothesis 4 (H4). Work engagement will be positively related to organizational identification.

Furthermore, this research extends the JD-R model by examining the influence of work engagement on resilience among officer cadets, a topic previously understudied. Unlike the traditional research direction, which often focuses on the impact of resilience on work engagement, this study hypothesizes the inverse relationship. Specifically, we propose that engagement, manifested through vigor, enthusiasm, and energy, is a critical determinant in enhancing resilience. Drawing on the principles outlined by Bakker et al. (2023), it can be inferred that engagement may lead individuals, such as cadets, to demonstrate a greater propensity to confront and endure challenging tasks. Such heightened engagement facilitates the development and effective use of personal and job resources, resulting in a “positive gain spiral” (Bakker, 2011). This dynamic process not only bolsters adaptability and the capacity to overcome difficulties but also promotes essential skills like advanced problem-solving, effective stress management, and fortified social networks within the organizational context. These elements are pivotal in cultivating resilience in demanding settings like military training. Based on these considerations, we propose the following hypothesis:

Hypothesis 5 (H5). Work engagement will be positively related to resilience.

**Figure 1:**
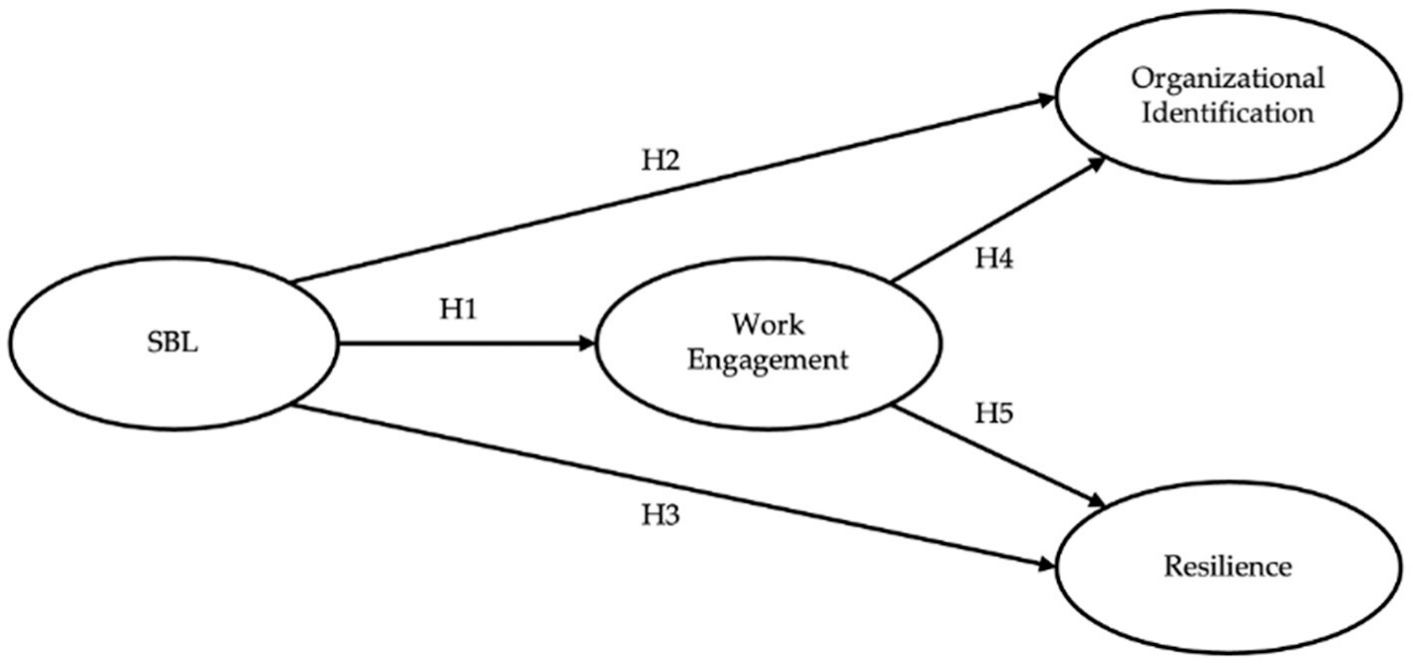
Research model. SBL, Secure Base Leadership.

Figure 1 outlines our theoretical model and hypotheses, offering a visual guide to the mediational process explored in this study. It illustrates the interplay between SBL and its effects on organizational identification and resilience, mediated by work engagement. On this basis, we propose the following mediation hypothesis:

Hypothesis 6 (H6). The impact of Secure Base Leadership on organizational identification and resilience is mediated by work engagement.

## 3 Materials and methods

### 3.1 Procedure and participants

This study is part of a larger research project funded by the Ministry of Science and Innovation of Spain, titled “The Leader as a Secure Base in the Military Context” (PID 2020-117780GB-100). Data collection was carried out in collaboration with the commanders of the General Military Academy of the Army in Zaragoza, Spain. The General Military Academy, incorporating the University Center of Defense affiliated with the University of Zaragoza, is the leading institution for higher military education in Spain, where cadets are trained to become officers in the Spanish Army. This program includes university-level academic studies in addition to general military training.

Prior to participation, the officer cadets were informed about the scientific objectives of the study and assured that their involvement was voluntary and anonymous. The participating cadets (*N* = 363) were primarily from the second (*n* = 170; 46.8%) and third (*n* = 193; 53.2%) years of their officer training. Their average age was 21 (SD = 2.4), with a gender distribution of 84% male and 16% female. They were required to evaluate the leader of their section (platoon chief captain) and complete self-report scales tapping their work engagement, organizational identification, and resilience. The average time of service under the assessed commander (leader) was 5.47 months (SD = 1.64), with 90% of these officers being male.

### 3.2 Measures

Upon obtaining participant consent, a questionnaire was administered to measure the following variables.

#### 3.2.1 Secure Base Leadership

Cadets' perceptions of their section chief captain as a secure base were assessed using the 15-item Leader as Secure Provider Scale (LSPS; Molero et al., 2019). Participants rated their agreement with each statement on a Likert scale from 0 (Strongly Disagree) to 4 (Strongly Agree), with items such as “When I need help at work, I turn to my leader.”

#### 3.2.2 Work engagement

This variable was measured using the three-item Spanish short version of the Utrecht Work Engagement Scale (UWES-3; Schaufeli et al., 2017). The items cover three dimensions: Vigor (e.g., “At my job, I feel full of energy”), Dedication (e.g., “I am enthusiastic about my job”), and Absorption (e.g., “I am immersed in my job”), rated on a Likert scale from 0 (Never) to 4 (Always).

#### 3.2.3 Organizational identification

The seven-item scale by Topa et al. (2009), adapted from Mael and Ashforth (1992) Organizational Identification Scale (OIS), was used. Responses were provided on a Likert scale from 0 (Strongly Disagree) to 4 (Strongly Agree). An example item is “When I talk about this organization, I usually say ‘we' instead of ‘they'.”

#### 3.2.4 Resilience

This variable was assessed using the five-item measure developed by Hardy et al. (2010) and validated in Spain by Valor-Segura et al. (2020). It measures the ability to maintain confidence in the face of challenges and dissatisfaction (e.g., “Bounce back from performing poorly and succeed”), with responses recorded on a five-point Likert scale from 0 (Low) to 4 (High).

### 3.3 Data analysis

In this study, descriptive statistics, including means, standard deviations, and correlations, were calculated using the SPSS software v.27. For further analysis, we employed Partial Least Squares Structural Equation Modeling (PLS-SEM). PLS-SEM is a non-parametric technique particularly effective for complex mediation models and for exploring advanced options such as the assessment of multiple mediators (Hair et al., 2017; Henseler et al., 2015). This method offers two key advantages for our study. Firstly, like other SEM techniques, PLS-SEM accounts for measurement error, providing more accurate estimates of mediation effects compared to regression analyses. Secondly, PLS-SEM is designed to handle smaller sample sizes and non-normal data distributions (Henseler et al., 2015). Analyses were conducted using SmartPLS v4.0 software (Ringle et al., 2024). Statistical significance was evaluated using the bootstrapping method with 10,000 samples of 363 cases, applying a critical *t*-value of 1.96 to determine significance at a *p* < 0.05 level. The model analysis was conducted in two phases (Hair et al., 2017). First, the reliability and convergent and discriminant validity of the measurement model were analyzed. Second, the hypothesized structural model was assessed, that is, to what extent SBL predicted organizational identification and resilience, considering work engagement as a mediator.

## 4 Results

### 4.1 Reliability and construct validity

In the initial phase of our analysis, we rigorously evaluated the validity, reliability, and internal consistency of the employed scales, including Cronbach's Alpha, Composite Reliability (CR), McDonald's Omega, and Average Variance Extracted (AVE). All the results exceeded the recommended cut-off values (Hair et al., 2017). Alpha, Omega, and CR coefficients exceeded the threshold of 0.70, and AVE values were >0.50 (see Table 1).

**Table 1:** Evaluation of the measurement models.

**Construct**	**Indicators**	**λ**	***t***	**α**	**ω**	**CR**	**AVE**
Work engagement	WE01	0.85	50.41^**^	0.78	0.80	0.87	0.70
	WE02	0.90	73.84^**^				
	WE03	0.75	20.24^**^				
Secure Base Leadership (SBL)	SBL01	0.60	14.51^**^	0.92	0.92	0.93	0.51
	SBL02	0.76	29.15^**^				
	SBL03	0.77	29.63^**^				
	SBL04	0.59	12.93^**^				
	SBL05	0.72	24.30^**^				
	SBL07	0.67	20.73^**^				
	SBL08	0.68	20.73^**^				
	SBL09	0.69	23.54^**^				
	SBL11	0.71	25.60^**^				
	SBL12	0.72	23.47^**^				
	SBL13	0.77	31.02^**^				
	SBL14	0.74	27.62^**^				
	SBL15	0.81	45.20^**^				
Organizational identification	OI01	0.81	28.17^**^	0.74	0.75	0.83	0.50
	OI02	0.64	12.93^**^				
	OI03	0.69	15.46^**^				
	OI04	0.65	9.49^**^				
	OI05	0.70	13.37^**^				
Resilience	R01	0.84	39.72^**^	0.86	0.85	0.90	0.64
	R02	0.79	28.58^**^				
	R03	0.70	19.33^**^				
	R04	0.84	36.44^**^				
	R05	0.83	44.61^**^				

^**^p < 0.01.

λ, factor loadings; t, t-statistics; α, Cronbach's alpha; ω, McDonald's omega; CR, composite reliability coefficient; AVE, Average Variance Extracted.

Our preliminary model comprised 30 indicators, collectively forming four latent constructs. Each indicator's reliability was assessed based on its factor loadings or correlations with the respective construct λ. It was anticipated that a factor loading of more than 0.60 for each indicator would be sufficient to effectively represent a latent variable, thereby accounting for a significant proportion of the variance (Hair et al., 2017). Our analysis revealed that the majority of the factor loadings across all scales were robust. However, two indicators from the SBL scale (Item 6 “I believe my platoon chief captain would support my growth and promotion at work” and item 10 “When I need help at work, I look for my platoon chief captain”) and two indicators from the organizational identification scale (Item 6 “I largely act as a typical member of my section” and item 7 “If the media criticized my section, I would be embarrassed”) fell short of this threshold and were consequently removed. This led to a refined model with 26 indicators, each exhibiting high factor loadings. The exclusion of these four items did not detract from the overall model's integrity, as shown in Table 1. Given the reflective nature of the model, a strong correlation among the indicators was maintained, with each indicator representing a unique aspect of the same underlying phenomenon.

Having established the reliability and internal consistency of our model's scales, we next turned our attention to evaluating discriminant validity. According to Fornell and Larcker's criterion, the Average Variance Extracted (AVE) values for each construct should surpass the squared correlations among the constructs to ensure adequate discriminant validity (Hair et al., 2017). This requirement is critical to confirm that each scale is distinct and measures unique constructs. In addition, the Heterotrait Monotrait Ratio (HTMT) serves as an additional check for discriminant validity. Henseler et al. (2015) suggest that HTMT values below 0.85 are indicative of acceptable discriminant validity among scales. Our model satisfactorily meets both these critical criteria for discriminant validity (see Table 2).

**Table 2:** Descriptive results, correlations, and discriminant validity.

	**Pearson correlation coefficients**						**HTMT**			
	***M***	***SD***	**1**	**2**	**3**	**4**	**1**	**2**	**3**	**4**
1. Work engagement	2.99	0.66	*0.83*				-			
2. Secure Base Leadership	1.87	0.77	0.37^**^	*0.71*			0.41			
3. Organizational identification	2.84	0.75	0.35^**^	0.34^**^	*0.70*		0.45	0.39		
4. Resilience	3.12	0.60	0.45^**^	0.23^**^	0.20^**^	*0.79*	0.26	0.54	0.20	-

1: Work Engagement; 2: Secure Base Leadership (SBL); 3: Organizational Identification; 4: Resilience. The diagonal elements marked in italics in the correlations are the square root of the Average Variance Extracted (AVE) for each construct.

^**^p < 0.01.

After establishing the reliability and validity of the measures, we utilized SmartPLS for the subsequent model fit assessment. This process incorporated both classic and modern indices. The Standardized Root Mean Square Residual (SRMR), a traditional measure of fit, showed a value of 0.067, indicating good model fit as values below 0.08 are generally considered acceptable (Hu and Bentler, 1999). In addition, we applied the unstandardized (d_ULS) and geomin (d_G) discrepancy measures. These indices assess the congruence between the proposed model and the observed data. The d_ULS values were 1.562 and 1.569, and the d_G value was 0.438, suggesting that the model adequately represents the data and therefore fits well (Ringle et al., 2024). Employing these indices enabled a comprehensive and robust evaluation of the model's fit.

### 4.2 Descriptive statistics and correlational analysis

Table 2 presents the descriptive statistics and Pearson correlation coefficients for the variables under investigation. Secure Base Leadership reveals a relatively low mean score (M = 1.87) but exhibits the highest standard deviation (0.77), signifying significant variability in scores among leaders in the respective sections to which the officer cadets belong. Conversely, the remaining variables in our study display relatively high scores, surpassing the midpoint of the Likert-type response scale set at 2. Notably, resilience stands out with the highest mean score (M = 3.12) and a lower standard deviation (0.60). Furthermore, we observed significant positive correlations between SBL and organizational identification (*r* = 0.34, *p* < 0.01), resilience (*r* = 0.23, *p* < 0.01), and work engagement (*r* = 0.37, *p* < 0.01). Additionally, work engagement exhibits positive correlations with organizational identification (*r* = 0.35, *p* < 0.01) and resilience (*r* = 0.45, *p* < 0.01). These findings align with our theoretical framework and offer initial support for our hypothesis.

### 4.3 Hypothesis testing

**Figure 2:**
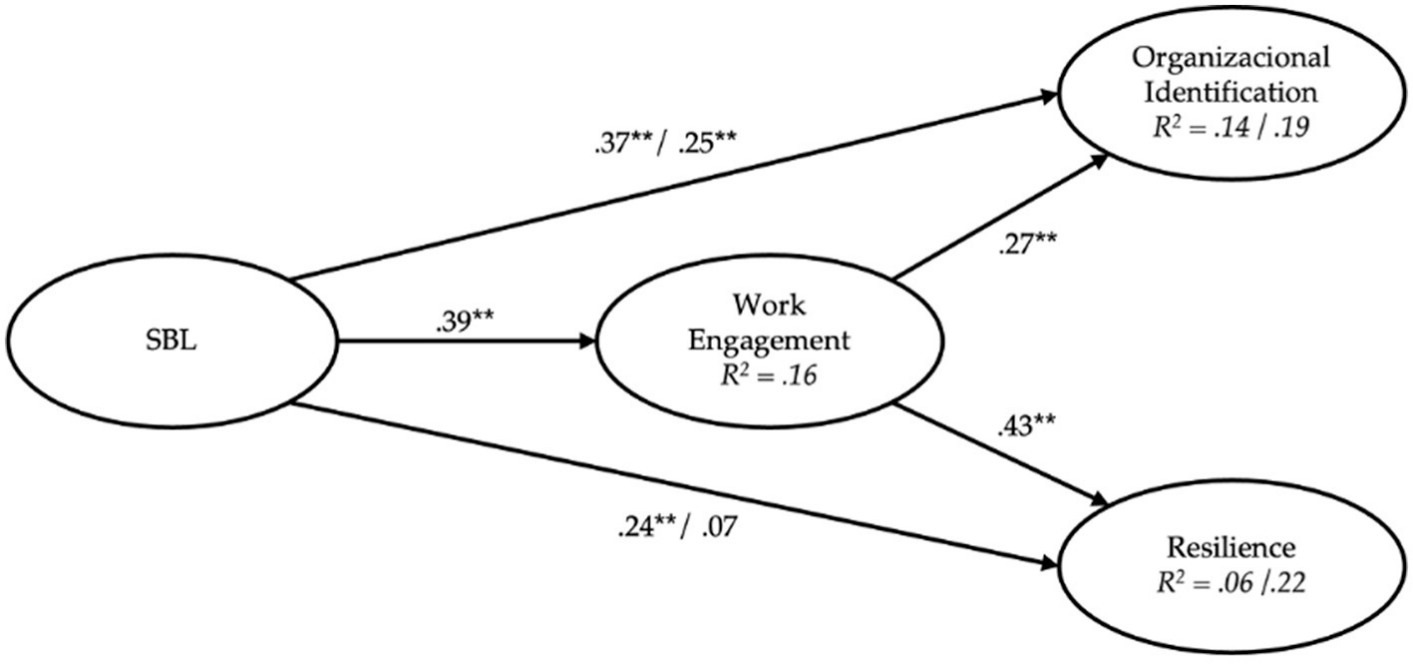
Standardized regression coefficients for the full structural model. SBL, Secure Base Leadership. Values preceding the “/” (slash) symbol denote standardized coefficients and explained variance for direct effects model. Values following the slash represent standardized coefficients and explained variance within the mediated model. **p < 0.01.

Figure 2 depicts the interplay between SBL and both organizational identification and resilience, scrutinized both directly and through the intermediary role of work engagement. The model assessing direct effects corroborates hypothesis 1, unveiling a positive and significant linkage between SBL and work engagement (β = 0.39, *p* < 0.01), which accounts for 16% of the variance in work engagement. In alignment with hypothesis 2, SBL had a statistically significant positive association with organizational identification (β = 0.25, *p* < 0.01). Hence, an elevation in SBL correlates with heightened levels of work engagement and identification with the organization among officer cadets. Regarding our hypothesis 3, we are observing a full mediation, as initially the relationship between SBL and resilience was significant (β = 0.24, *p* < 0.01). However, this direct effect disappears when the mediating variable, work engagement, is included (β = 0.07, *p* = 0.20). Moreover, work engagement is shown to have significant direct relationships with both organizational identification (β = 0.27, *p* < 0.01) and resilience (β = 0.43, *p* < 0.01), supporting hypothesis 4 and 5, respectively.

The examination of indirect effects, as presented in Table 3, provides empirical validation for Hypothesis H6. First, the indirect positive effect of SLB on organizational identification mediated by work engagement was statistically significant (β = 0.10, *p* < 0.01). However, because the direct effect of SLB on organizational identification was still significant after controlling for work engagement (see Figure 2), we can speak here of a partial mediation through work engagement. Second, the indirect positive effect of SLB on resilience mediated by work engagement was also statistically significant (β = 0.16, *p* < 0.01). In this case, because the direct effect of SLB on resilience was no longer significant after controlling for work engagement (see Figure 2), we can speak here of full mediation of work engagement. This in-depth analysis highlights the critical mediating role of work engagement in the relationship between SBL, organizational identification, and resilience.

**Table 3:** Structural equation model and hypothesis test results.

	**Estimate**	**SD**	***t*-value**	**Hypothesis**
**Direct effects**				
SBL -> Work engagement	0.39	0.05	8.38^**^	H1 accepted
SBL -> Organizational identification	0.37	0.05	8.00^**^	H2 accepted
SBL -> Resilience	0.24	0.05	4.75^**^	H3 accepted
WE -> Organizational identification	0.27	0.06	4.21^**^	H4 accepted
WE -> Resilience	0.43	0.06	7.76^**^	H5 accepted
**Indirect effects**				
SBL -> Work engagement -> Organizational identification	0.10	0.03	3.67^**^	H6 accepted
SBL -> Work engagement -> Resilience	0.16	0.03	5.21^**^	

SBL, Secure Base Leadership; WE, Work Engagement.

^**^p < 0.01.

## 5 Discussion

The primary aim of this study was to examine the role of work engagement in mediating the contributions of Secure Base Leadership to organizational identification and resilience. According to the JD-R model, work engagement functions as a pivotal mediating construct, bridging the gap between job-related demands and personal resources, and subsequently influencing a spectrum of organizational and personal outcomes (Bakker and Demerouti, 2007). More specifically, we have posited a theoretical model (Figure 1) wherein Secure Base Leadership (SBL), as a fundamental job-related resource rooted in attachment theory, exerts its influence on work engagement, which, in turn, contributes to both organizational identification as an organizational-level outcome and resilience as a personal-level outcome.

Our findings support the proposed hypotheses. A significant and positive association was identified between SBL and work engagement in officer cadets at the Spanish General Military Academy, aligning with the JD-R model and supporting Hypothesis 1. This underscores SBL's integral role as a vital job resource, fostering motivation and enhancing engagement levels. Furthermore, SBL's nurturing and empowering effects are found to considerably strengthen organizational identification among the cadets, thereby supporting Hypothesis 2.

The study further revealed a statistically significant direct effect of SBL on resilience, in line with the expectations of Hypothesis 3. Nevertheless, it is noteworthy that SBL accounts for merely 6% of the variance in resilience. This limited explanatory power may be attributed to the demographic characteristics of our sample, which includes relatively young cadets at the preliminary stages of their military careers. Given that resilience is a characteristic that evolves and strengthens over time, reflecting personal development and maturity, it is plausible that these early-stage cadets have not yet faced sufficiently challenging experiences to significantly influence or assess their resilience (Masten, 2001; Pietrzak and Southwick, 2011).

Our analysis also supports Hypothesis 6, suggesting that work engagement acts as a mediator between SBL and both organizational identification and resilience. Specifically, we found a partial mediation in the relationship between SBL and organizational identification, indicating that while SBL directly influences organizational identification, work engagement also plays a significant mediating role. We also found that the relationship between SBL and resilience is characterized by full mediation through work engagement, meaning the contribution of SBL to resilience is entirely conveyed through its positive impact on work engagement, without a direct effect of SBL on resilience itself.

Incorporating the JD-R model within the military academy context enriches our understanding of motivational dynamics critical for military education and leadership development (Bartone et al., 2007; Bates et al., 2013). According to the JD-R model, high engagement occurs when provided resources effectively counterbalance the job-related demands cadets might face during their military training. These resources include supportive leadership, a constructive organizational culture, and role clarity. Collectively, these resources assist cadets in addressing challenges, advancing their personal development, and enhancing their learning. This model highlights a mutually beneficial relationship between resources and engagement within the military academy context, where resources derived from a security-enhancing leader not only enable cadets to effectively engage with military tasks but also promote their sense of belongingness and personal development (heightened organizational identification and resilience).

Moreover, it is important to emphasize that SBL should not be regarded as just another leadership style. Instead, we prefer to view it as a common factor that underpins the diverse range of positive leadership models (e.g., transformational leadership, empowering leadership, and servant leadership). The overarching objective of SBL is to foster interpersonal relations in which individuals feel both close and free, aligning perfectly with attachment theory's concept of a secure base that facilitates exploration and growth with the confidence that support will be available when needed.

By integrating the JD-R model and attachment theory, our study provides a comprehensive understanding of the intricate interplay between leadership, resources, engagement, and personal development within the context of military academies. This holistic approach underscores the significance of not only addressing job demands but also nurturing a supportive and secure environment that empowers cadets to excel in their roles, identify with the organization, and develop resilience. Our study contributes valuable insights to the broader discourse on leadership in high-stress environments and offers practical implications for leadership development programs within military education.

While our study sheds light on several important aspects, it is essential to acknowledge its limitations. First, the cross-sectional nature of this research limits our ability to infer causality. While high correlations between leadership and work engagement have been noted (Gutermann et al., 2017; Pastor et al., 2019), longitudinal studies are needed to better understand this relationship in military contexts. Second, the use of self-report measures, albeit with adequate psychometric properties and validated in Spain, may introduce response biases. Future research should thus consider mixed methods approaches to triangulate findings. Finally, our study focused on subordinates' perceptions of their supervisors as secure bases. Future research could explore the long-term effects of SBL on cadet development and its applicability in other high-stress professional environments. Subsequent studies could also incorporate supervisors' self-assessments to provide a more valid understanding of Secure Base Leadership and its impact on burnout in military environments. Additionally, further investigation into the interaction between different leadership styles within the JD-R model could provide a more nuanced understanding of employee engagement in various organizational contexts.

The view of SBL as a significant job resource within the JD-R framework not only carries practical implications for leadership training and development in military academies but also highlights the theoretical nuances of applying such models across different organizational contexts. This finding underscores the imperative for leadership styles that adeptly address job demands while furnishing the requisite resources to foster engagement, resilience, and organizational identification. From a theoretical standpoint, it suggests the potential for extending the JD-R model, traditionally applied in civilian organizations, to military settings, thereby enriching our understanding of leadership dynamics across diverse organizational landscapes. Furthermore, the role of work engagement as not merely an outcome but also a precursor in the relationship between leadership and resilience adds a compelling layer to the discourse, echoing the reciprocal relationships discussed in the theoretical framework. This nuance invites a deeper exploration of the bidirectional nature of engagement within leadership paradigms, offering both theoretical and practical insights into the development of more resilient and engaged military personnel.

## 6 Conclusion

In conclusion, the integration of SBL within military academies represents a significant paradigm shift in leadership approaches. It emphasizes the pivotal role of supportive and responsive leadership in enhancing work engagement, organizational identification, and resilience among cadets. Our study not only validates the JD-R model within a military context but also highlights the transformative impact of SBL on cadets' professional growth and wellbeing.

## Data Availability

The raw data supporting the conclusions of this article will be made available by the authors, upon reasonable request.
